# Supplementary material for: GRT-X Stimulates Dorsal Root Ganglia Axonal Growth in Culture via TSPO and Kv7.2/3 Potassium Channel Activation
Source: Int J Mol Sci. 2024 Jul 3;25(13):7327. doi: 10.3390/ijms25137327 (PMC11242890; doi:10.3390/ijms25137327)
Supplement: Supplementary file 1 [file ijms-25-07327-s001.zip › ijms-3046811-supplementary/Supplementary Material word.pdf]

Supplementary Material

Supplementary Material S1A:

Coverslips' coating:

Circular plastic coverslips (**LFG distribution 174950**) were incubated with poly-L-lysine (**Sigma P7890**) at a final concentration of 0.05mg/mL in 0.15M Sodium Borate Buffer for 30 mins. After 3 washes (5 minutes each) with PBS 1X, the coverslips were incubated 30 minutes with collagen (**R&D systems 3440-100-01**) diluted (1:7) in acetic water, in the presence of 35% Ammonia. After collagen removal, the resulting thin layer allowed DRG adherence to the coverslips.

Supplementary Material S1B:

Media compositions :

- DRG Plating Media

Component	Final Concentration	Reference
DMEM high glucose	Base medium	11960044 Gibco
Glutamax (100X)	1X	35050061 Gibco
Heat inactivated Horse Serum	10%	26050088 Gibco
2.5S NGF	50ng/ml	1156NG R&D
Antibiotic/ Antimycotic (100X)	1X	15240062 Gibco

- Neurobasal Media

Component	Final Concentration	Reference
Neurobasal Medium	Base medium	21103049 Gibco
Glutamax (100X)	1X	35050061 Gibco
2.5S NGF	50ng/ml	1156 NG R&D
B27 supplement (50X)	1X	17504044 Gibco
Antibiotic/ Antimycotic (100X)	1X	15240062 Gibco

Supplementary Material S1C : Primer sequences

Primer name	Sequence
**Tspo Forward**<br>**Tspo Reverse**	GCCTCCGGTGGTATGCTAG<br>GCGTCCTCTGTGAAACCTC
**Kv7.2 Forward**<br>**Kv7.2 Reverse**	CGTGACTATCGTGGTATTCG<br>GCTTCCTGGCAAACTTGAG
**Kv7.3 Forward**<br>**Kv7.3 Reverse**	CCCAGTCAAGAGGAACAACG<br>ACGGCCAGAATCAAGCATC
**Mpz Forward**<br>**Mpz Reverse**	ATCTCTTTTACCTGGCGCTACC<br>ACTGGATGCGCTCTTTGAAG
**Mbp Forward**<br>**Mbp Reverse**	ACTCACACACGAGAACTACCC<br>GGTGTTCGAGGTGTCACAATG
**Dhh Forward**<br>**Dhh Reverse**	ATCCACGTATCGGTCAAAGC<br>AGTCACCACGATGTAGTTCCC
**Plp Forward**<br>**Plp Reverse**	AGCAAAGTCAGCCGCAAAAC<br>CCAGGGAAGCAAAGGGGG
**Krox20 Forward**<br>**Krox20 Reverse**	TGCACCTAGAAACCAGACCTTC<br>TGCCCGCACTCACAATATTG
**CNPase Forward**<br>**CNPase Reverse**	ACTCCTTACCTCACGCCATC<br>AGCATTGCTGTTGGACTTGG
**Cad19 Forward**<br>**Cad19 Reverse**	TGACATAGGGGAGAATGCAGAG<br>AAGCTCTTCATCCACATGGC
**Tfap2α Forward**<br>**Tfap2α Reverse**	ACTCCTTACCTCACGCCATC<br>AGCATTGCTGTTGGACTTGG
**Stmn2 Forward**<br>**Stmn2 Reverse**	AGGACGAGGGTAAAGAAGAG<br>TTGCCACACAGAGAGAAAG
**Peripherin Forward**<br>**Peripherin Reverse**	GGTGCTCTTCCGTAAGGAC<br>GCACCTGCTGGCTCTCTAC
**Nfh Forward**<br>**Nfh Reverse**	AAGCACCCCACAGACATCAG<br>CCTTGGGAGCTTCGTCTTTC
**GAPDH Forward**<br>**GAPDH Reverse**	ATCCCATCACCATCTTCCAG<br>CCATCACGCCACAGTTTCC
