# Supplementary figures and images for: GRT-X Stimulates Dorsal Root Ganglia Axonal Growth in Culture via TSPO and Kv7.2/3 Potassium Channel Activation
Source: Int J Mol Sci. 2024 Jul 3;25(13):7327. doi: 10.3390/ijms25137327 (PMC11242890; doi:10.3390/ijms25137327)

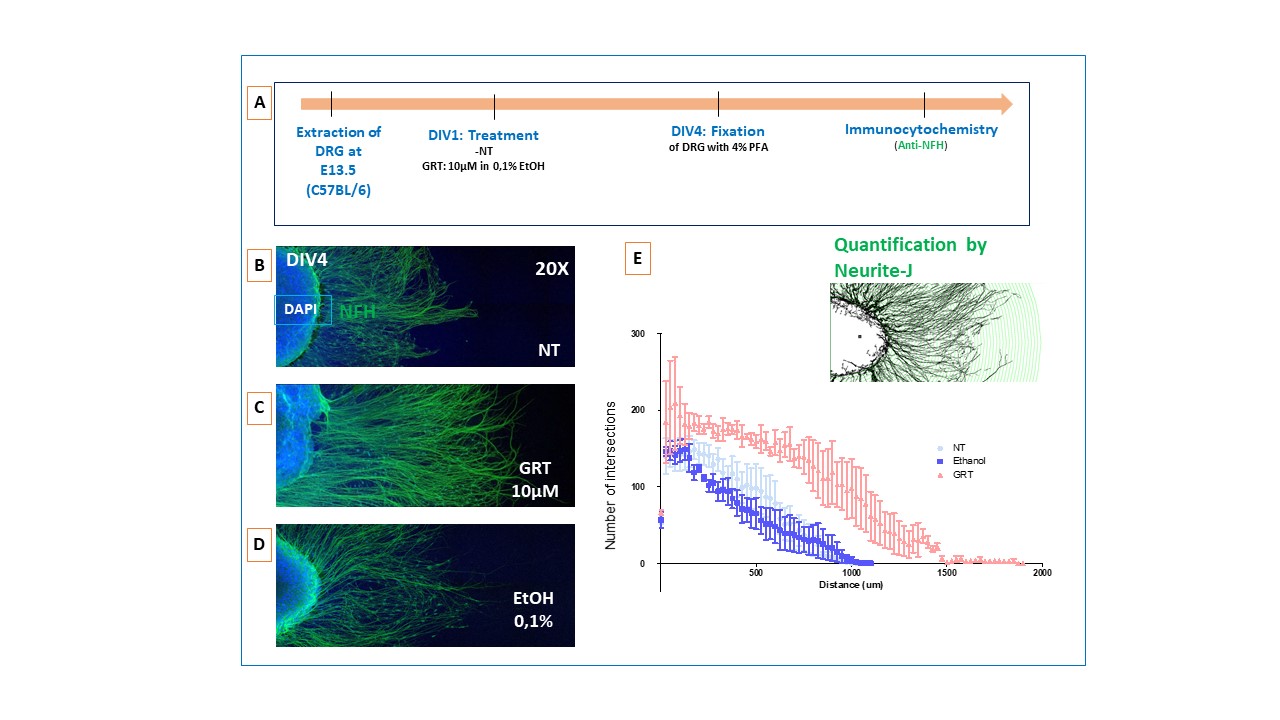

Supplement: Supplementary file 1 [file ijms-25-07327-s001.zip › ijms-3046811-supplementary/Supplementary material final/Supplementary FIgure S1 GRT-X effect on axonal growth.jpg]

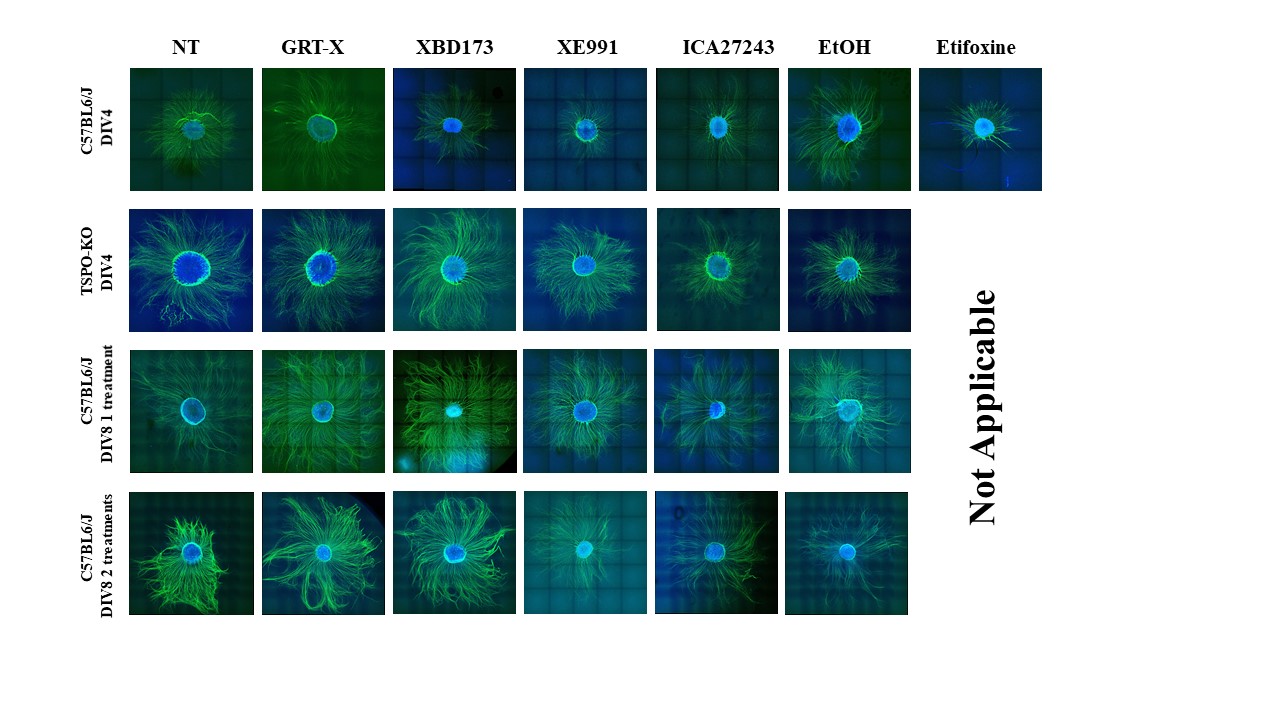

Supplement: Supplementary file 1 [file ijms-25-07327-s001.zip › ijms-3046811-supplementary/Supplementary material final/Supplementary Figure S2.jpg]
